# Sample matching by inferred agonal stress in gene expression analyses of the brain

**DOI:** 10.1186/1471-2164-8-336

**Published:** 2007-09-24

**Authors:** Jun Z Li, Fan Meng, Larisa Tsavaler, Simon J Evans, Prabhakara V Choudary, Hiroaki Tomita, Marquis P Vawter, David Walsh, Vida Shokoohi, Tisha Chung, William E Bunney, Edward G Jones, Huda Akil, Stanley J Watson, Richard M Myers

**Affiliations:** 1 Stanford Human Genome Center, Stanford University, Palo Alto, CA, USA; 2 Department of Genetics, Stanford University, Palo Alto, CA, USA; 3 Molecular & Behavioral Neuroscience Institute, University of Michigan, Ann Arbor, MI, USA; 4 Center for Neuroscience, University of California, Davis, CA, USA; 5 Department of Psychiatry & Human Behavior, University of California, Irvine, CA, USA

## Abstract

**Background:**

Gene expression patterns in the brain are strongly influenced by the severity and duration of physiological stress at the time of death. This agonal effect, if not well controlled, can lead to spurious findings and diminished statistical power in case-control comparisons. While some recent studies match samples by tissue pH and clinically recorded agonal conditions, we found that these indicators were sometimes at odds with observed stress-related gene expression patterns, and that matching by these criteria still sometimes results in identifying case-control differences that are primarily driven by residual agonal effects. This problem is analogous to the one encountered in genetic association studies, where self-reported race and ethnicity are often imprecise proxies for an individual's actual genetic ancestry.

**Results:**

We developed an Agonal Stress Rating (ASR) system that evaluates each sample's degree of stress based on gene expression data, and used ASRs in *post hoc *sample matching or covariate analysis. While gene expression patterns are generally correlated across different brain regions, we found strong region-region differences in empirical ASRs in many subjects that likely reflect inter-individual variabilities in local structure or function, resulting in region-specific vulnerability to agonal stress.

**Conclusion:**

Variation of agonal stress across different brain regions differs between individuals, revealing a new level of complexity for gene expression studies of brain tissues. The Agonal Stress Ratings quantitatively assess each sample's extent of regulatory response to agonal stress, and allow a strong control of this important confounder.

## Background

Comparing cases and controls is one of the most widely used methods in genetic and epidemiological research to identify disease risk factors at the population level. From the study design standpoint, to maximize the power of detecting a true effect it is important to understand the major sources of phenotypic variation, and to minimize sample heterogeneity accordingly. Furthermore, to reduce the number of spurious positive findings due to confounding factors, it is important to match cases and controls on "well-established determinants" [1] that are not themselves the variables of direct interest. In practice, however, it is often difficult to declare *a priori *which variables, out of many that are examined, are the *established *risk factors. Occasionally, the major factors affecting the phenotypic outcome may be truly strong and well known, such as cigarette smoking as a risk factor for lung cancer, or older age for Alzheimer's disease. In most other situations, however, particularly those concerning multifactorial diseases such as cancer and psychiatric disorders, there are usually numerous contributing factors for the observed phenotype, but their relative importance is not always known beforehand. While many case-control studies automatically include age and gender in sample matching, additional variables that are important for the phenotype need to be chosen on a case-by-case basis, and sometimes only after the data have been collected and analyzed. In genetic association studies, for example, the ancestral background of human subjects can have a strong confounding effect [for example, [2-5]]. A parallel situation exists for gene expression analyses involving the use of postmortem samples, where tissue pH and near-death physiological stress can exert a major influence on the inter-individual variation of expression patterns [6,7]. The impact of pH/agonal stress is so strong that it often far outweighs the influence of all other factors, including age and gender, and can obviate the detection of the impact of the illness. Because of this, more and more gene expression studies in recent years take special precaution to match samples by pH and agonal factors, just as a well designed genetic study seeks to balance cases and controls by self-reported racial identity or continental ancestry.

Despite the widespread use and general success of these sample matching strategies, the risk of residual confounding remains. The key pH-sensitive genes or pathways, such as components of mitochondrial electron transport chain and proteasome genes, are highly variable between samples [8], and often appear as the top findings in microarray-based case-control comparisons of brain samples. For example, while several studies have reported down-regulation of mitochondrial transcripts in schizophrenia [9] and bipolar disorder [10], others have reported up-regulation [11,12]. The samples used by Prabakaran et al. [9] had a slight case-control difference in pH, but many more controls than cases died of cardiac events [13]. Further analyses of the same RNA samples suggested that most of the findings that implicate mitochondria genes could be explained by effects of medication. Interestingly, the samples used in Sun et al. [10] were not balanced in pH, although the clinical condition appeared to be balanced. These conflicting results suggest that the role of pH-sensitive, stress-related genes in psychiatric disorders is still unresolved. Similarly, in the parallel example of genetic association studies, several recent analyses have highlighted the need for more stringent controls for the very strong genetic confounders [3,4]. In studies involving highly diverse populations, many human subjects are admixed at the individual level, that is, they carry genetic material derived from several founding populations. For such individuals, a single self-reported racial or ethnic descriptor such as "African American" or "Hispanic" is no longer adequate for representing the proportional contribution from multiple ancestral origins. It has become more desirable, and in fact feasible, to infer the individual admixture ratios from the observed genetic data [14-16], and to apply these empirically derived ratios in *post hoc *sample matching [17,18]. In effect, what was initially a sample classification problem, in which subjects are categorized into discrete ethnic groups, has been turned into a "grades of membership" problem [19], in which individual samples are scored on one or several continuous variables. These continuous variables can be used for sample selection, case-control matching, or as a new covariate in regression analyses, stratified analyses, or ordered subset analyses [20-22]. The empirically derived ancestral ratios may be more effective for mitigating the impact of confounding, because they can be more accurate than self-reported ancestry, as the former are derived from the genetic data *per se*, and are less susceptible to survey errors or recall bias.

In this study, we applied a similar strategy to an ongoing gene expression study in which we compare postmortem brain tissues between normal controls and subjects who suffered from major depression, bipolar disorder, or schizophrenia. In a previous report [6], we described a classification-based analysis in which gene expression patterns in most subjects can be assigned to one of two main types: one from a low-pH, highly stressed group of samples, named "Type 2", and the other from a normal-pH, low-stress group of samples, named "Type 1". These two prototypes of expression patterns can be distinguished by strong and systematic changes in several biological pathways, including genes involved in energy metabolism and stress response. Since that report, we have increased the scope of our investigation from three brain regions in 40 subjects to six regions in up to 126 subjects (some regions were studied in fewer than 126 subjects). In carrying out case-control analyses, we found that even among the supposedly "purified" subset consisting of only the Type 1 samples, some residual heterogeneity in pH/agonal stress may still be driving the case-control comparison results, largely because of the overwhelming impact of agonal stress. Meanwhile, the pH- and stress-related genes that we and others have characterized continue to appear in the literature as among the top gene expression findings in comparative studies for a variety of diseases and conditions [9,23]. This experience motivated us to seek finer control of this obscuring variable by characterizing sample heterogeneity in greater detail. Specifically, we refined our previous dichotomous classification scheme to one that evaluates group-membership by quantitative ratings. A second rationale for pursuing this study came from the recognition that pH values are typically measured in one or two brain regions (in our case, cerebellum), whereas disease-related changes in gene expression are expected to occur in numerous brain regions. There is no *a priori *reason to assume that altered pH and agonal factors would impact all these brain regions in a uniform manner. Consequently, sample matching based on a parameter derived from a single brain area or the entire brain may not be reliable for all regions examined, whereas gene expression data for individual regions can be used to assess specific regional patterns of agonal stress.

To this end, we developed Agonal Stress Rating (ASR), a quantitative system that measures the degree of stress of each RNA sample on a continuous scale based on gene expression data. We examined the relationship between ASRs and conventional *pre hoc *indicators such as pH and clinically derived Agonal Factor Scores (AFS), compared the stress ratings across six brain regions, and assessed the performance of different sample matching strategies. We also developed rigorous data pre-processing methods, compared different options of defining the ASRs, evaluated the robustness of ASRs in terms of the between-lab and between-platform reproducibility, and explored several related analysis algorithms.

## Results

### Systematic technical variation and data processing strategies

Before we begin to characterize biological confounding factors, non-biological sources of variation must be identified. The microarray data used in this study were collected in multiple experimental batches, representing the mixed use of two types of Affymetrix Genechips (U133A/B and U133Plus_2), experiments run at three laboratories (at UC Irvine, UC Davis, and University of Michigan), RNA samples from six brain regions (AnCg, DLPFC, AMY, HC, CB, and NACC), and six cohorts of approximately 20 subjects each (four Mood Disorder Cohorts and two Schizophrenia Cohorts), for up to 126 subjects, about half of which were normal controls, the other half were cases of major depression, bipolar disorder, or schizophrenia. Cohort assembly, tissue dissection and RNA extraction took place in multiple stages, typically several months apart. The RNA samples were labeled and hybridized one cohort at a time, one region at a time, in two or three laboratories (called "Sites", not to be confused with the six brain regions) separately. As a result of these technical variabilities, the entire dataset contained systematic differences between sites, chip types, and sometimes, cohorts, although the cohort-cohort technical differences are blended with genuine sample-sample differences across cohorts. This type of technical variation warrants careful scrutiny, and must be adequately controlled to ensure the accuracy of analyzing biological differences.

**Figure 1:**
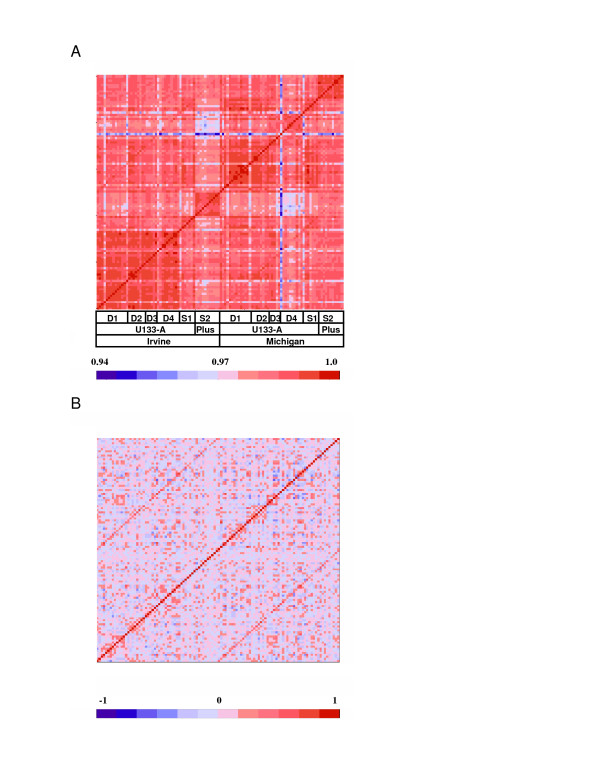
**Technical "block" effects due to cohort, chip-type, and laboratory (site)**. a. A color-coded correlation matrix among 201 AnCg samples. Red indicates high correlation between pairs of samples; blue indicates low correlation. Samples were ordered by technical batches, which in turn were defined by Cohort, Chip Type, and Site, as indicated below the heatmap. Throughout this paper the sample order in correlation heatmaps is the same from left to right as from bottom to top. Pearson's correlations were calculated by using all U133A transcripts. Rectangular "blocks" of high correlation, indicated in red, correspond to samples that are highly similar to each other in gene expression patterns. These block-block partitions coincide with the natural boundaries of experimental batches as indicated below the heatmap. D1 through D4 indicate Depression Cohorts 1 to 4, while S1 and S2 indicate Schizophrenia Cohort 1 and 2. b. The same correlation matrix after removing the block effect by median centering each block. The diagonal line of high values are self-self correlations, but the two off-diagonal lines of relatively high correlation values are between replicate chips for the same samples ran at two sites. The sample order and block partitions are the same as in 1a.

After array scanning and Affymetrix Genechip data summary (a computational process that combines data from multiple oligonucleotides probes designed to interrogate a given transcript to obtain a single expression value for that transcript, see Methods for more details), we examined chip-to-chip similarity in each region by plotting the pairwise correlation matrices as color-coded heatmaps, where red indicates high similarities between pairs of chips, blue indicates low similarity, and the samples are ordered by site and by cohort. Figure 1a showed one such correlation map for 201 AnCg chips produced by using logged intensities of all 12,734 Refseq gene-based probe sets on the U133A chip. In this example, as is the case for other brain regions, we analyzed data from two sites in our Consortium for six cohorts, representing 12 naturally occurring experimental batches. Figure 1a shows that the observed patterns aggregate in rectangular "blocks" of high correlation, indicated in red, corresponding to samples that are highly similar to each other in gene expression patterns. Importantly, the block-block partition coincides with the natural boundaries of experimental batches. Not all experimental batches can be definitively separated from each other; typically the 12 batches can be adequately described by 5–9 blocks, as sometimes two adjacent cohorts from one site form a single indistinguishable group, mostly due to relatively homogeneous technical conditions shared across these cohorts. In all, block-like structures are seen in every brain region, and almost always correspond to experimental batches, suggesting that they arose from changes in reagents, hybridization protocols, chip types, or scanning conditions.

At least two other lines of evidence suggest that the "blocks" are derived from technical variation between experimental batches rather than due to genuine biological differences between samples in different cohorts. First, when we set aside data for all human transcripts, and plot chip-chip correlations by using only the 68 Affymetrix control probe sets, which target spiked-in *E. coli *transcripts, the data still exhibit the same block structure as seen with the use of all genes [see Additional File 1, figure 1a], indicating that technical factors play a major role in delineating the blocks. Secondly, when we re-ran all samples on a custom 711-gene Illumina Beadarrays in a validation experiment that was done at one site and randomized samples across cohorts and regions, we did not observe the block-like separation between cohorts [see Additional File 1, figure 1b], suggesting that biological differences between cohorts made a minor contribution, if any at all, to the observed "blocks".

The correlation matrices not only provide a means to visualize sample heterogeneity, but also allowed us to define a most parsimonious set of blocks for each region for the purpose of data normalization. To adjust for the block effect, we subtracted from each sample's logged expression value the median value of the block, and did so for each block and for every transcript. For example, for each gene, expression values for all Block 1 samples would subtract the median value of Block 1, and likewise for all other blocks, such that after the centering, the median of each block is at zero, effectively transforming the original data into the deviations from the block medians, in a procedure that is similar to adding a Block factor as a categorical variable in robust linear modeling (particularly the median polish method). The goal of this procedure is to remove a block-wide fixed factor, most of which, as we argued above, came from technical sources. The benefit of this adjustment, particularly the assumption of a fixed block-specific effect, can be evaluated by an objective criterion: how well the adjusted data increase the technical reproducibility of the same samples that were run at two or more sites. We found that after removing the block effect by median centering, we improved the between-site similarity for replicate chips run at multiple sites (Figure 1b). For samples that were run on both the U133A and the U133Plus_2 chips, removing a fixed between-chip-type effect produced satisfactory agreement between the two chip types [see Additional File 1, Figure 1c].

Although the systematic differences between blocks can be adjusted in this way, the assumption of a fixed effect is not expected to hold for all genes in all samples equally well. Other types of variation, including within-block heterogeneity, however, are not readily discernible in the data, and are probably impossible to control. Our analysis showed that a major portion of the between-block differences have been accounted for by a fixed technical effect affecting all samples within the block, for most of the genes studied. In the example of 201 AnCg chips, the median proportion of variance explained by the "block" factor, across all 12,734 genes, is 2.5% of the total variance. Because the 2.5% between-block variance has one degree of freedom, while the 97.5% within-block variance has 199 degrees of freedom, the F test showed that more than half of the genes had significant between-block differences. In this case, 56% of the genes satisfy P < 0.05 (one-way ANOVA) for the Block factor.

### Biological variation across samples

**Figure 2:**
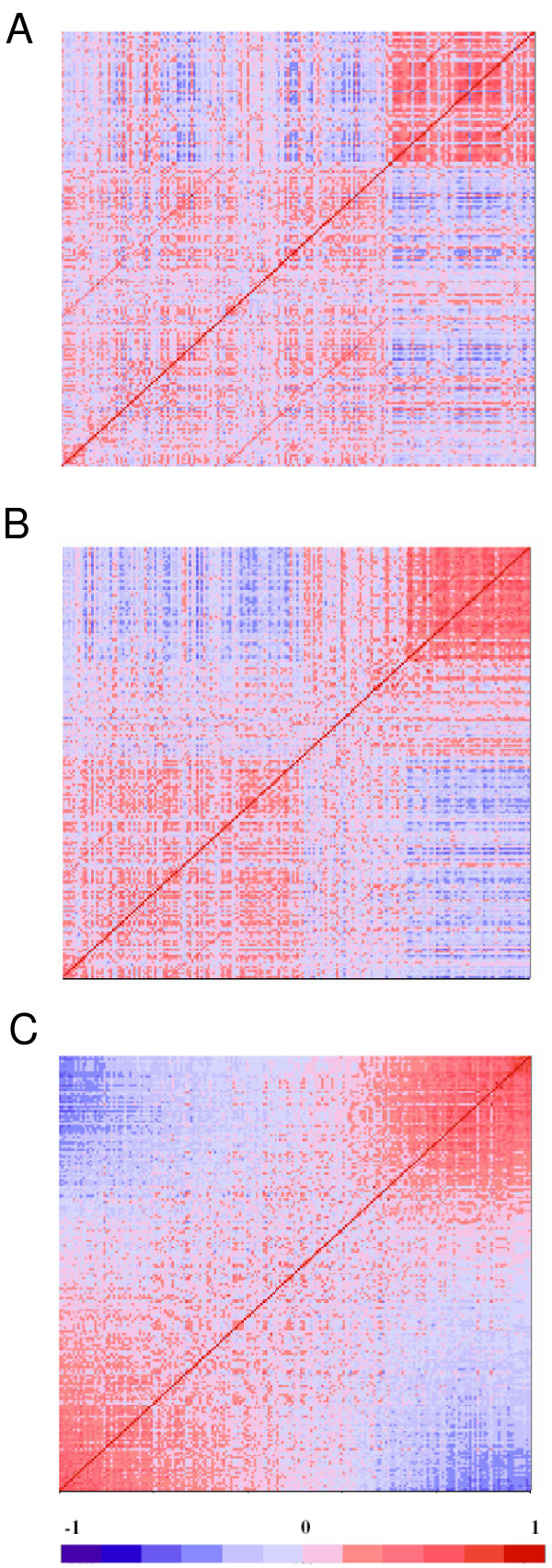
**From discrete classification to grades of membership**. a. The same correlation matrix as in Figure 1b but with samples re-grouped so that the Type 1 samples are on the lower left side, showing that samples can be classified into two main types of expression patterns. b. The same samples can be subdivided into three classes, and re-ordered accordingly in the correlation heatmap, still showing reasonably strong distinction between the three classes. c. The same heatmap re-ordered by the first Principal Component scores, resulting in a gradual progression from one prototype to the other.

Previously we showed that most of the brain samples in our study can be classified into two main types of expression patterns [6]: those from individuals who died quickly and had normal tissue pH, and those from individuals who suffered prolonged death, typically with medical complications, and exhibited low tissue pH. The threshold value between low and normal pH is around 6.6 in our samples, but it varied among different studies and different tissue collections [24]. Figure 2a shows the correlation heatmap for the 201 AnCg samples obtained by using expression values already adjusted for the block effect as described above. This is the same plot as in Figure 1b, but with the samples re-ordered so that the previously designated Type 1 samples are grouped to the lower left side of the graph. While the distinction between the two classes can be clearly seen, there are still samples of intermediate patterns that may correspond to varying degrees of agonal stress that do not readily belong to the two opposing prototypes.

Because pH/agonal stress acts as an exceedingly strong confounder in gene expression studies, a dichotomous classification may not be sufficient to ensure that cases and controls are well-stratified, or balanced within each stratus. In addition, different brain regions may carry different stress outcomes; whereas this aspect of data heterogeneity is not well informed by pH values measured in one brain region, nor by the clinical indicators. One possible approach for dealing with residual heterogeneity is to subdivide the main classes further, such as into Types 1A, 1B, 2A, 2B, etc, effectively classifying samples in multiple tiers to establish nested subsets, within each of which the cases and controls are more homogeneous and better balanced. Figure 2b shows an example of three-type classification; however, even finer subdivisions are clearly possible. In practice, it is difficult to decide the number of clusters or layers of clusters needed, and the samples often show genuinely graduated differences of expression patterns. A natural alternative to a finer-grained classification approach, therefore, is to rate samples on one or several continuous scales. Toward this goal, we first carried out a Principal Component Analysis. We re-ordered the 201 AnCg sample by their first principal component (PC1) scores, and the resulting heatmap (Figure 2c) shows a gradual transition from one end of the spectrum to the other. Similarly smooth progressions are also observed for the other five regions (not shown). Importantly, the PC1 scores are highly correlated with pH (r = 0.59 for AnCg, P < 10^-13^), the clinical agonal factors, as well as with the previously determined Type 1-Type 2 designations; whereas PC2 and PC3 scores have almost no correlation. For example, in AnCg, r is 0.04 (P > 0.35) and 0.07 (P > 0.25) for pH-PC2 and pH-PC3, respectively. Note that with n = 126, r needs only to be approximately 0.18 to be significantly non-zero at p = 0.05). This result not only confers a biological meaning to PC1, but also suggests that a single continuous variable is likely to be sufficient to capture most of the gradual progression of expression patterns from the low-pH prototype to the normal-pH prototype.

**Figure 3:**
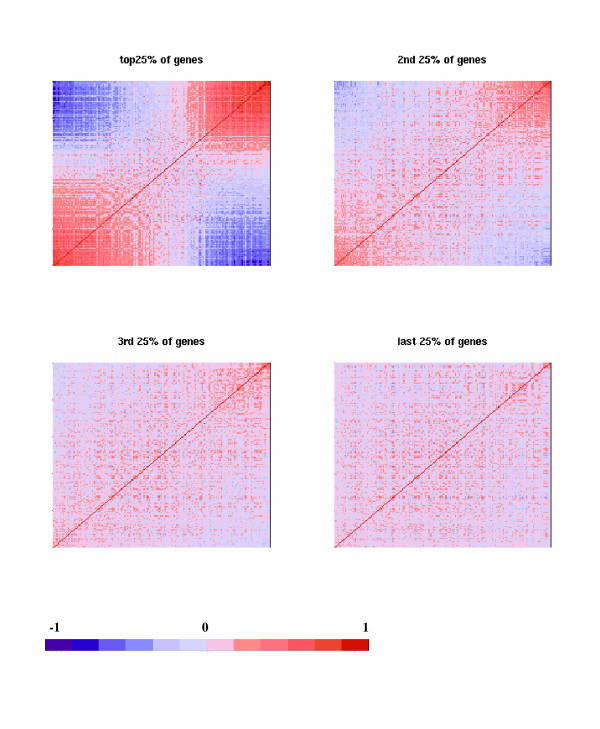
**Informativeness of genes for agonal stress**. The same heatmap as shown in 2c but with the correlations calculated by using the 25% strongest Type 1-Type 2 genes (upper left), the next 25% (upper right), the third 25% (lower left), and the last 25% of genes (lower right). The genes were ranked by comparing 40 samples (~20% of the total of 201 samples) on the lower left side in 2c against 40 samples on the upper right side in a Student t test.

Not all genes contributed equally to the placement of individual samples along the gradient of membership. We selected the 20% strongest Type 1-like samples (on the lower left corner of Figure 2c) and the 20% strongest Type 2-like samples (on the upper right corner), and calculated the Student's t scores that contrasted the group means between these two canonical groups. This allowed us to rank genes by their Type 1- Type 2 absolute t scores, with the "top genes" being those that are most strongly affected by pH/agonal stress. When we re-plot Figure 2c by using the 25% strongest pH-sensitive genes (instead of all 12,734 genes), a strong gradient is clearly seen (Figure 3, upper left panel). The gradient between samples becomes much dampened when the second 25% of ranked genes (upper right of Figure 3) is used, and fades away almost entirely with the use of the third and last 25% of ranked genes (lower panels of Figure 3). This result indicates that the top 50% of genes are likely to be informative for membership inference, with the top 25% and top 5% of genes carrying increasingly greater discriminating power, as one would expect. In Additional File 2 we showed a heatmap of expression levels of the top 25% of transcripts across 201 AnCg samples. These genes have been used to calculate the sample-sample correlations shown in Figure 3, upper left panel. The genes are ordered from left to right by their coefficients in the first principal component (i.e., the first eigenvector), whereas the samples are ordered from top to bottom by their first principal component scores. The actual expression levels are provided in Additional File 3, which is a .cdt file that can be opened in Java Treeview for flexible browsing. To estimate the number of genes significantly affected by the pH effect, we used the Nearest Shrunken Centroids classifier [25] to calculate the cross-validation errors in a two-class classification analysis, and examined gene panels containing varying numbers of most discriminating genes. Panels having as few as 297 (2.3% of the total of 12,734 probe sets) and as many as 4,720 (37.1%) genes resulted in eight or fewer cross-validation errors out of 201 samples, and formed a plateau of error curve [see Additional File 4], indicating a broad range of the number of informative genes. Similarly, in genetic association studies, some DNA polymorphisms are more informative for distinguishing different populations [26]. In our procedure to construct an Agonal Stress Rating for individual samples (described below), we used 25% or 5% of "top genes", and always found that with the 5% top genes the intermediate ratings are more "stretched out" than with 25% of top gene (not shown), as one would expect, because the strongest pH-sensitive genes are more powerful in distinguishing subtle differences in intermediate grades of membership. We ran the Principal Component Analysis for six regions separately and defined the strongest Type 1/2-like samples for each region. When we subsequently compared the strongest Type-1 against the strongest Type-2 samples and ranked genes by their t scores, the top 5% or 25% genes are similar across the six regions as their t scores are highly correlated across regions [see Additional File 5]. On average, a top (or bottom) 10% gene in one region has a 57% chance to be among the top (or bottom) 10% in another region. We ranked genes by their average t score ranks in five regions (all except CB, as cerebellum is an outlier region for gene expression due to its unique anatomical and physiological properties), and listed the 1000 most strongly up-regulated and 1000 most strongly down-regulated genes in Additional File 6. Genes in these lists can be used as most informative genes in future, independent studies.

### Agonal Stress Ratings

Because the PC1 scores successfully arrange the samples into a smooth gradient, these scores by themselves could serve as a measure of agonal stress in individual samples. However, we found that PC1 may be strongly influenced by the scale of variance in individual chips and sometimes by a small number of "outlier" samples, whereas our method of using PC1 as a crude criteria to pre-select the strongest ~20% Type 1- and Type 2-like samples is more robust to these outliers. In addition, the interpretation of the Principal Component scores requires the notion of decomposition of the observed gene expression patterns into the linear combination of multiple components. This interpretation is most natural in cases where each sample represents the actual mixing of multiple cell types, each having its own canonical expression patterns. The meaning of PC scores is also complicated by the process of analyzing log-transformed signal values, while actual transcript levels are "mixed" on the linear scale. Alternative indices, such as the probability of classification [25], or the prediction strength such as the "margin-of-victory" measures adopted in Golub et al. [27], are most appropriate when there are multiple genuinely discrete outcomes, for which what is uncertain is the strength of evidence for class assignment. In our case, gene expression in the brain is most likely affected by agonal stress in a graded (albeit non-linear) fashion. Although many of the samples belong to the two extreme states–one is minimally affected by stress, the other for samples that have converged to the quasi-steady state of "thoroughly affected"–some tissues in our collection are apparently sampled at an intermediate physiological state, and can best be characterized by the sample's relative distance to the two ends of the spectrum, that is, the distance to the prototypical normal-pH pattern minus the distance to the prototypical low-pH pattern, where "distance" can be the Pearson's correlation, Euclidean Distance, Spearman's rank correlation, or a number of other metrics. The Agonal Stress Rating is thus defined as the difference between each sample's distances to the two prototypical patterns, and can be calculated by using different sets of most informative genes, which in turn can be defined by comparing the most extreme samples (see Methods). Although ASR is formally neither a probability of classification nor a mixing ratio, it is actually quite similar to these other measures as most of them are variants of the linear discriminant function.

**Figure 4:**
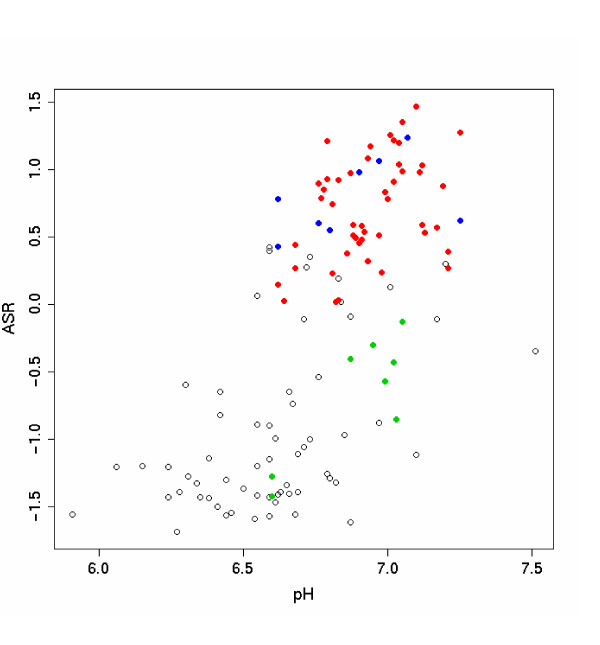
**Correlation of ASR and pH values**. Median ASR values across 2–3 sites and six brain regions for 126 subjects versus their brain pH values. Samples indicated in red and black were in general agreement between pH and ASR. The eight samples in blue were previously removed from analysis due to either low pH or some clinical complications (AFS is non-zero), but had normal ASR values and likely had not experienced significant agonal stress. The eight samples shown in green were previously included but had low ASR values, and should better be removed in order to reduce sample heterogeneity and possible case-control imbalance.

**Figure 5:**
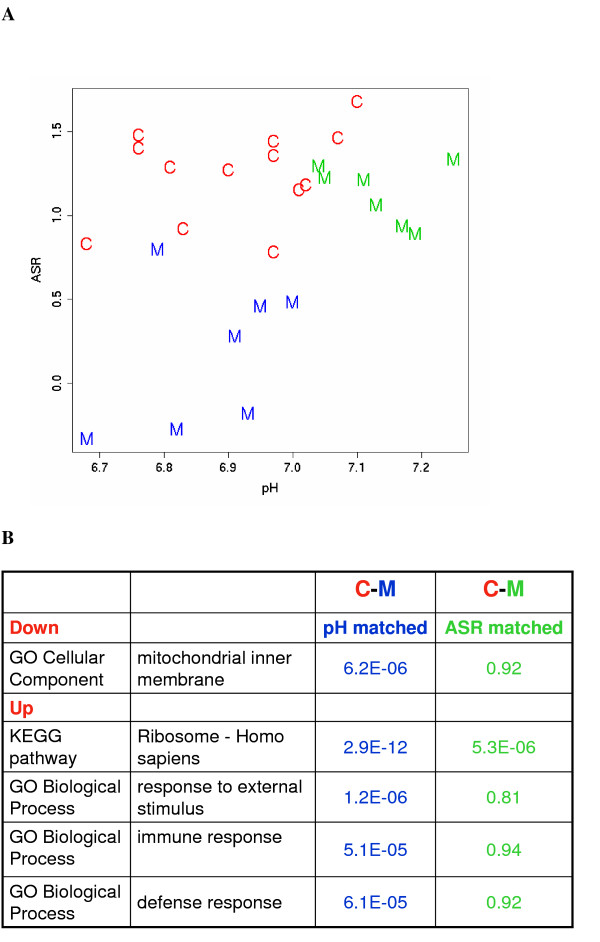
**Comparing pH-matching and ASR-matching with an example**. a. ASR and pH values for samples used in a test case, where a group of pH-matched major depression samples (in blue) were compared to controls (in red), while a second group of ASR-matched major depression samples (in green) were compared to the same controls. b. In an analysis of enrichment of Gene Ontology and KEGG pathway terms for the top genes, the pH-matched comparison still revealed significant effects for gene families and pathways known to be affected by the pH effect, while the same effect was much attenuated in the ASR-matched comparison.

A comparison of the median ASR values (for each subject, across regions and sites) with tissue pH (Figure 4) reveals two features. First, there is a general correlation, i.e., low pH samples tend to have low ASRs. Secondly, there is considerable local discrepancy, i.e., among the normal pH samples, the ASR-pH correlation is weak. These discrepancies imply that the ASR values, which are derived directly from the gene expression data, are sometimes at odds with the measured brain pH. In the face of such discrepancies, we need to determine which index is a more accurate surrogate for the actual degrees of stress experienced by the individual subjects in specific brain regions, and therefore provides a safer control of the agonal stress confounder. We examined a test case: from a set of 55 AnCg samples previously "included" in an intermediate-stage analysis, we selected all of the thirteen Control samples with ASR > 0.8, and divided all of the fourteen Major Depression samples into two groups (Figure 5a): one that was matched with the 13 Controls in pH (6.91 +/- 0.13 in controls, 6.87 +/- 0.11 in cases, p = 0.45, t test) but not in ASR (1.25 +/- 0.27 in controls, 0.18 +/- 0.44 in cases, p = 0.0003), another matched to the Controls in ASR (1.25 +/- 0.27 in controls, 1.14 +/- 0.18 in cases, p = 0.28) but not in pH (6.91 +/- 0.13 in controls, 7.13 +/- 0.08 in cases, p = 0.00015). We carried out MDD-Control comparisons for the two MDD groups separately, and analyzed the top and bottom 4,000 genes in EASE (the Expression Analysis Systematic Explorer) [28] for significantly enriched Gene Ontology terms. Figure 5b shows that the pH-matched comparison yields the gene families and pathways associated with agonal stress that we and others previously discovered, whereas the ASR-matched group significantly reduces the effect. This result is not surprising, as by the definition of ASRs, we effectively have balanced the key stress-related pathways when we match samples by ASR. This test case, however, shows that matching by pH is not always safe for guarding against the agonal stress confounder, whereas the empirically derived ASR values provide a more accurate assessment of the regulatory responses to near-death stress in individual samples, and allow a more stringent control. In a section below we will describe the robustness ASRs, particularly the finding that deriving ASRs by using only the control samples did not substantially change the result.

### Between-region differences

**Figure 6:**
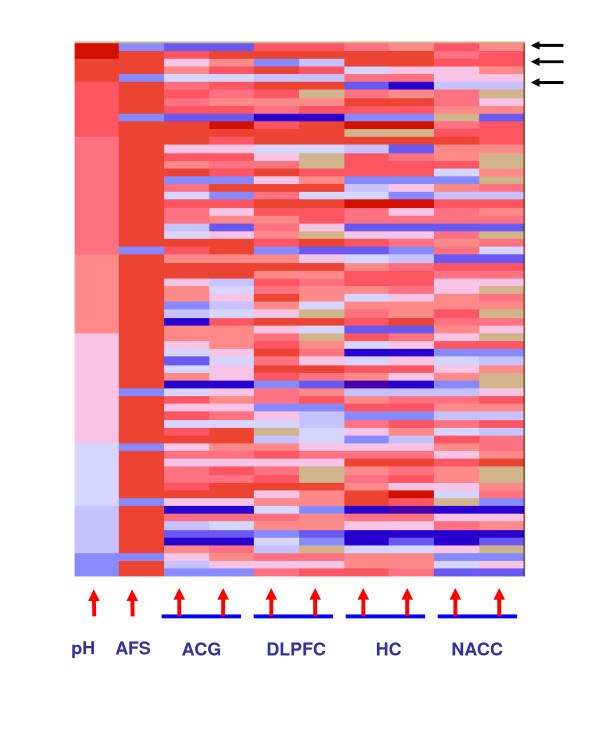
**Region-region difference of ASRs**. A color-coded table of (from left to right) pH values (red is higher pH), AFS scores (0 complication in red, AFS = 1 in light blue), and eight series of ASR values (red-higher ASR) for 68 subjects satisfying pH>6.6 and at most two missing values out of eight ASR series. The eight ASR columns represented four brain regions, each measured in two sites. Tan-colored elements indicate missing data because the samples were not run.

After calculating ASR for all six regions and for two sites in each region, we obtained twelve series of ASR values. As AMY and CB had larger numbers of missing chips (samples not analyzed on microarrays), we plotted the eight ASR series for the four remaining brain regions in color codes along with pH values and AFS (in two levels) (Figure 6, see figure legend for color codes), with the samples sorted by pH (high pH on the top of the figure), and missing data in white. This figure has several broadly recognizable features. First, ASR results from the two sites tend to agree, although not always so. Secondly, ASRs in most samples tend to be correlated across the four regions, reflecting brain-wide patterns. Thirdly, the ASR scores show a coarse correlation with pH and the clinical AFS scores, as samples at the bottom of the figure are generally those with both low pH and low ASR (this can also be seen in Figure 4, where pH is plotted against the median ASR across the six brain regions). The correlations between pH and ASR, across all 126 samples, range from 0.3 to 0.6 among the twelve ASR series (from 0.57 to 0.63 in AnCg, DLPFC, CB, and NACC, 0.47 in HC, and 0.3 in AMY, which has the smallest sample size: 66 out of 126 total samples. Note that pH was measured in CB). But these correlations are much smaller when using only the 90 pH>6.61 samples, indicating that much of the correlation was driven by the large differences between the high-pH and the low-pH samples. Finally, some of the subjects who had normal pH yet low ASR can be explained by having clinically recorded agonal stress (AFS = 1 or higher).

However, beyond these large trends, there remain striking between-region differences in many individual subjects. For example, the first subject from the top, indicated by the first arrow on the right, has low ASRs (in green) in AnCg, and normal to high ASRs in the other regions. The third and sixth subjects, indicated by the next two arrows, have unusually low ASRs in DLPFC and HC, respectively. These strong region-region differences in ASRs are robustly observed (see the next section) and suggest genuine differences in stress outcome across different parts of the brain. Such differences may arise due to inter-individual variabilities in local structure or function, resulting in region-specific vulnerability to agonal stress. An additional possibility is that the nature of the illness that caused the death, such as hepatic, renal, or cardiac failures, may have triggered a region-specific brain response. These results underscore the value of using ASRs to carry out per-region sample matching, as pH and clinical indicators are measures for the entire brain.

### Robustness of ASRs

It is difficult to know how much between-region differences arise from variabilities in tissue dissection, but this factor is unlikely to explain most of the differences we observed here, as the effect is seen even for relatively large, easy-to-dissect regions such as DLPFC and CB. Although the nature of the observed regional differences is still not clear, most of such differences are likely due to actual biological differences rather than to technical variability in performing RNA labeling and hybridization or to data analysis methods. There are several reasons for making this conclusion. First, while regional differences of ASRs are often large (+/- 0.5), ASRs varied to a much lesser degree (1) between sites, (2) between different numbers of informative genes used, (3) between different distance measures (correlation, rank correlation, or Euclidean distance), (4) with another round of normalization after median centering, (5) following the selection of only the high ASR samples as the basis for re-calculating block medians, or (6) importantly, following the use of only normal controls rather than both cases and controls for ranking genes by t scores. Secondly, we examined the possibility that some regions may actually have a greater Type 1-Type 2 difference than the other regions, and different samples may have different regional differences. To this end, we calculated additional versions of ASR by using (1) the same ten Type 1 samples and the same 14 Type 2 samples to determine the informativeness of genes in all six regions; (2) combined all six regions (more than 1200 chips) in designating the canonical samples on the two extremes, defining most informative genes, and calculating ASR across all 1200 samples (all regions together). These alternative versions did not significantly alter the ASR patterns across samples and regions, and did not explain most of the observed regional differences. Thirdly, with our 700-gene Illumina data, we were able to compare the ASRs based on data from two different platforms. For each sample, we averaged the Affymetrix ASR values from two sites to obtain the six values for six regions, and calculated their correlation to the corresponding six ASR values from the Illumina data. We obtained 67 such correlations in 67 samples for which we had both the Affymetrix and Illumina data. Of these 67, 58 correlations were above 0.33, 43 were above 0.67, and 28 were above 0.8, with most of the smaller correlations explained by very low between-region variations, that is, when the six scores are similar to begin with for a given sample, it is more difficult to observe a highly correlated pattern for this sample in a different platform.

## Discussion

Many gene expression analyses have shown that agonal stress at the time of death can significantly alter expression patterns in postmortem tissues [29-33]. Because of this, comparative studies that rely on postmortem material must take every precaution to ensure that cases and controls are properly balanced with regard to this well-established confounder. What remains unclear, however, is to what extent residual imbalance still accounts for the most significant findings. The study presented here is motivated by two considerations. First, a dichotomous classification of good-quality versus bad-quality samples is often inadequate in assuring that cases and controls are well matched among the "good samples". Secondly, clinical variables such as AFS and continuous variables such as tissue pH have several practical limitations, both in terms of accuracy and in their inability to assess each brain region separately. To address these concerns, we developed a rating metric to infer agonal stress based on expression data, and strongly advocated a conservative approach that uses these empirical ratings to select a homogeneous group or to balance cases and controls. The adoption of continuous ratings instead of the more commonly used categorical assignment is expected to bring several advantages: providing a more reliable measurement of underlying heterogeneity that does not show natural boundaries, retaining important information regarding sub-threshold variability, and allowing flexible integration with other variables.

### Between-region differences

Our results revealed striking between-region differences in stress outcome. This complexity presents additional challenges to gene expression analyses of the brain, especially if the goal is to not only study each region separately, but also to study biological regulation across brain regions, as well as their disruption in the diseased state. During our initial analysis, we had hoped that all regions of the same brain would have a similar stress profile, making it possible to use one or more regions as the same-subject control for the other regions. In this scheme, between-region differences in gene expression, instead of gene expression levels in each region separately, are compared between cases and controls, under the assumption that agonal stress is acting more extensively across the entire brain, whereas expression signatures of psychiatric disorders are restricted in some regions. This approach is reminiscent of the genetic method of using family controls to remove the impact of population structure. Our analysis, however, revealed considerable heterogeneity between brain regions in stress-related expression patterns, suggesting that this between-region analysis method is still problematic and requires further investigation.

### The need for a conservative approach

One of the concerns in adopting this approach is that gene expression changes in response to near-death conditions may overlap with those affected by chronic psychiatric states. If the Agonal Stress Ratings reflect the blending of both effects, how do we know whether we have under- or over-corrected the main effect of interest? Unfortunately, at the present time we do not know many genes or pathways that are clearly and reproducibly altered by major depression and bipolar disorder; as a result we currently do not have a definitive set of "true positives" to fine-tune the stringency of the sample matching strategy. To address this type of question would require a sufficiently large collection of samples to directly investigate whether patients of mental disorders are more likely than the healthy controls to have more severe terminal stress, lower tissue pH values, and more pronounced changes in gene expression patterns. When the sample size is below or near 100, as is currently the norm in expression profiling of human brains, a study is not properly powered to formally answer this question, and the gene expression signature due to mental disorders, if it overlaps with that due to agonal stress, will be easily obscured. However, despite this uncertainty, it is widely recognized that the effects due to agonal stress usually have a much more potent influence on gene expression patterns than do mental disorders. In such a situation, the chance of finding false positives due to residual imbalance is quite high. A continuous, quantitative rating system, like the ASR that we developed here, can greatly reduce the number of false positives in such studies by allowing a quantitative assessment of the broadly recognizable outcome of the most prominent confounder. By applying the ASR, it remains possible to detect a true signal for psychiatric disorders as long as it is strong enough or sufficiently independent of the confounding effect, particularly if the disease related changes involve a small number of transcripts whereas the agonal conditions usually affect a large number of genes [6]. In essence, the practice of sample matching by data-derived indices constitutes a higher standard for a "true positive", requiring that its main effect (for psychiatric disorders) to rise above its potential colinearity with the major extraneous covariates (changes due to agonal stress). The situation is analogous to the case of a genetic association study of, for example, cardiovascular disorders, where the goal is typically to detect genetic association with the disease while adjusting for the traditional risk factors, such as blood pressure, diabetes, body-mass index, and serum levels of HDL and triglyceride [34]. Because these traditional risk factors are also influenced by genetic variation, making such corrections constitutes a more focused goal: to discover genetic contribution to the disease *independent of the genetic basis of the other risk factors*, even though in the long run it is ideal to be able to study all the contributing factors in conjunction with each other. By the same token, the near-term goal of our study is to discover gene expression signatures of psychiatric disorders independent of gene expression changes due to confounding factors, especially when these factors (stress at death) are not likely to be closely related to the biology of mental illness.

### Conventional versus empirical indicators

In this study, we found that the Agonal Stress Ratings are sometimes at odds with tissue pH values or clinically-recorded medical factors. These indices, though useful in most situations, have several shortcomings when serving as surrogates for the underlying biological heterogeneity. First, different brain regions may carry different stress outcomes, while pH values were usually measured in one region. Likewise, the clinical indicators do not inform regional heterogeneity. Secondly, these conventional variables inevitably contain measurement errors or incomplete information. Thirdly, brain pH is both the outcome of prior episodes of stress and the trigger for subsequent physiological responses. As a result, even if pH is measured in all regions, and even if pH is similar across regions [32], the samples that have similar pH are still not likely to have a uniform stress-induced gene expression profile. For these reasons, we recommend using ASR as a primary criterion in sample selection and sample matching, analogous to using inferred ancestry in genetic association studies [15].

It is also important to point out that the tissue pH measurements and clinical records are still of immense value in sample matching. They provide biological meaning to the ASR values, and allow an integrated strategy that combines these different indicators in sample selection [35]. We believe that future studies need to continue collecting clinical information as extensively as possible, and if feasible, measure pH in multiple brain regions to directly assess regional differences of degree of stress.

### Assumption of neutrality

An implicit assumption of using inferred ancestry to control population stratification in genetic studies is that most of the genetic markers tested are phenotypically neutral; that is, not associated with the disease under study. Under this assumption, when data have been collected for a large number of loci (that is, not just for a few candidate genes), the majority of these loci can be used to infer population structure [14,16,36,37]. Alternatively, they can be used as Genomic Control loci to detect over-dispersion of the standard test statistics due to stratification [38-40]. This assumption of neutrality generally holds when we expect to detect large or moderate signals of association for only a small number of causative genes. In gene expression analysis, however, it is possible that a considerable proportion of the transcripts are affected both by the extraneous confounders such as agonal stress and by the disease being studied. As a result, some of the genes used to infer agonal stress may also be associated with the disease, making it difficult to separate the two effects. However, this apparent difference between genetic and gene expression studies depends on the actual context of the study. Population stratification can be a very strong confounder for traits or diseases that have subtle genetic effects but large population differentiation (in incidence rates as well as in allele frequencies), or are studied in recently admixed population where chromosomal segments of defined ancestry can be as long as 5 cM [41]. In these situations, a substantial fraction of genetic markers may show weak association, while most of such signals may disappear after correcting for inferred individual ancestry or locus-specific ancestry. For both gene expression and genetic studies, using the data *per se *to detect sample heterogeneity is a practically useful yet ultimately exploratory method, especially when true positives are not known beforehand.

### Model considerations and the need for canonical patterns

In inferring individual ancestry from genetic data, it is helpful to know the genetic profile of the putative ancestral populations, for example, the allele frequencies determined from samples that represent the progenitors of the admixed individuals. Without these "pure" canonical profiles, while it is still theoretically possible to simultaneously determine both the canonical ancestral patterns and the mixing ratios of each sampled individual, the results are less reliable and often improperly scaled [16]. Similarly, our strategy to derive Agonal Stress Ratings relies on having obtained data for both "normal" tissues and severely stressed tissues. These opposing extremes serve as the two reference points to measure the grades of membership in between. Hypothetically, in a different study, if none of the samples (or too few of them) are from the severely stressed low-pH samples, the canonical signature of the confounder (or the principal components) would be much less precisely defined. However, even when the absolute values of ASR only scale from "normal" to mildly stressed, the relative ratings among samples can still be used as the empirical basis for sample matching, or as the basis for a variety of *post hoc *strategies, such as stratified analysis, or incorporating covariates in regression analysis. We have found that the main differences due to agonal stress can be robustly observed, i.e., are highly similar between study sites and across two microarray platforms. This means that the canonical patterns that we describe here, featuring prominently energy metabolism genes and stress response pathways, can be transferred to other studies of postmortem tissues even if these studies characterized a narrower range of the confounding effect. In Additional files 3 and 6 we provide the genes most strongly affected in our dataset; these genes can be used as informative stress markers for pre-evaluating future sample collections, even before microarray experiments. The parallel example in genetic studies is that one can profile the genetic signature for a few major continent groups (such as in the International HapMap Project) and use this information repeatedly to infer admixture in individuals from a heterogeneous population such as the African Americans, sometime by using a small number of most informative genetic markers [26]. We also wish to emphasize that using only the controls to define the two canonical expression patterns did not change the ASR ratings in any meaningful way, reflecting the fact that the case-control differences are far subtler than the stress-related differences.

The situation would be much simpler if the mixing ratios are known by other, independent methods, or if the canonical patterns were profiled in independent studies. Such an "expression signature bank" can be established by studying the response to controlled stress treatment in animal models, or by analyzing "pure" classes of brain cells, such as laser-captured defined cell populations. Stuart et al. [42] gave an example where the mixing ratio is known for tumor samples when tissue pathologists reported *a priori *the fractional composition of different cell types in each sample. Conversely, Lu et al. [43] used expression data for synchronized yeast cells in defined phases to infer mixing ratios of asynchronized samples–this is a case where the basic patterns were known, with mixing ratios being the target of inference. When neither is known, as in our case, the solution cannot be arrived at analytically but can be optimized iteratively. For example, several methodology studies [44-46] have implemented variants of the Expectation-Maximizing (EM) algorithm to simultaneous search for the unknown canonical patterns and the unknown mixing ratios in gene expression data. Similar methods, including ones involving Markov Chain Monte Carlo methods, have been developed for inferring individual's genetic ancestry [14,16,47]. In our dataset, as the principal pattern is relatively strong, we expect the result to converge quickly and decide to adopt a simple definition of ASR. In more complicated situations, for example, when the second or higher Principal Components can be interpreted as signatures of other confounders such as age, medication, or cell types, it would be important to apply these more sophisticated algorithms.

## Conclusion

In this study we developed an Agonal Stress Rating (ASR) system that evaluates each sample's degree of stress based on gene expression data, and used ASRs in *post hoc *sample matching. We found that ASR-based matching provides tighter control of the agonal effect than by using pH. We also found that different brain regions exhibited different stress outcomes and that such regional patterns also varied between individuals. Our results once again highlight one of the main challenges in gene expression studies of psychiatric disorders: transcript levels are under the influence of a large number of confounding factors. Agonal stress undoubtedly plays a major role; as a result the stress-related, pH-sensitive genes or pathways are prone to appear as the top findings in case-control comparisons. We propose to adopt a conservative approach for the genes and pathways that are clearly altered by the confounding factors, even if it is possible that they are also influenced by the disease of interest. Deriving an ASR from gene expression data and using it for sample matching is one example of such an approach. This approach involves using continuous ratings as opposed to categorical assignments, thus represents an early attempt to apply graded classification methods to a sample heterogeneity problem for brain tissues, similar to the application of dimensional models in psychiatric diagnosis. In practice, we believe that this method can and should be expanded to the characterization of other sources of biological variation, such as medication, age, and cell-type composition of the dissected brain tissues. Drug use by psychiatric patients is likely to affect gene expression, regardless whether the medication was effective in treating the disease or not. But medication history is one of the most elusive aspects of clinical information to accurately record and quantify. Likewise, an individual's chronological age may vary greatly from the actual physiological age of the tissue under study, while the neuronal-glial ratios of the dissected tissues may also vary from brain region to brain region, from subject to subject. Proper monitoring of these additional sources of phenotypic variation is an important prerequisite for the eventual identification of the true gene expression signature of mental disorders, and in this regard, animal models of either drug treatment or stress, independently applied, in the absence of psychiatric history and genetic heterogeneity, represents a powerful alternative strategy for defining canonical expression patterns and assessing their relative contributions.

## Methods

Sample acquisition, RNA labeling, and microarray hybridization were carried out as described previously [6,48]. We used RMA [49] to obtain the probe set summary values for the Affymetrix U133A and U133Plus_2 Genechips. We removed chips that were clearly outliers, or failed the "gender-test", in which we confirm that Y-chromosome transcripts are only detected in male samples. The entire dataset, consisting 1218 Genechips, has been deposited to the Gene Expression Omnibus [50] with the Accession Number GSE6306.

Since the original Affymetrix probe definitions require frequent updates, and are known to contain errors and redundancies, we developed a custom probe definition method that involves re-annotating all Affymetrix probes by sequence alignment to the most recent build of genomic DNA sequences and a variety of transcribed sequence collections, including Unigene, Refseq, ENSEMBL Genes/Transcripts/Exons, and Entrez, etc [51]. For each of these transcript definition systems, the probes that could be uniquely assigned to their transcript targets were assembled in custom Channel Definition Files (CDF files) as individual probe sets, which assumed the names of the matched transcripts, thus replacing the Affymetrix probe set ID's. The analysis presented here used the third generation of our Unigene-based CDF files, which were based on Unigene Build 176. The CDF files can be downloaded for free at [52].

We calculated chip-chip correlation in each region by using RMA summaries values and defined blocks by visual inspection of the correlation heatmaps. Logged expression values for each gene were centered within each block. Median centered values were used for the Principal Component Analysis, whereby the PC1 scores were used to rank samples so that a proportion of samples at one extreme were designated canonical Type 1 samples, while a proportion of samples at the other extreme were designated canonical Type 2 samples. These two groups of samples were compared by the Student's t test, so that all genes can be ranked by the t scores. The ASR of each sample is then calculated as the "distance" to canonical Type 1 pattern minus its distance to the canonical Type 2 pattern by using a proportion of the genes with the largest absolute t scores. A range of parameter values were tested, including different proportion of samples used to define canonical patterns, different number of genes used for calculating ASRs, and different measured of chip-chip distance. For example, most results in this report were based on using approximately 20% of samples at each extreme of PC1 scores, 25% of the genes of largest t scores, and Pearson's correlation as distance measures. The nearest Shrunken Centroid Classification was carried out by using the Predictive Analysis of Microarrays (PAM) package in R. All R scripts used in the analysis are available from the authors.

The Illumina custom Beadchips were designed to cover ~700 transcripts that represent both biologically candidate genes and preliminary Affymetrix results to be validated. Sample labeling and hybridization were performed according to manufacturer's specifications. In all, about 67 samples were analyzed in each brain region, except for AMY, which we analyzed only 54 samples. All of the nearly 400 RNA samples were randomized with regard to cohort and region. The calculation of ASRs was carried out in a similar fashion as with the Affymetrix data except that there were only 700 genes used in the analysis.

## Abbreviations

AnCg, anterior cingulate cortex; DLPFC, dorsal lateral prefrontal cortex; AMY, amygdala; HC, hippocampus; CB, cerebellum; NACC, nucleus accumben

## Competing interests

The authors are members of the Pritzker Neuropsychiatric Disorders Research Consortium, supported by the Pritzker Neuropsychiatric Disorders Research Fund L.L.C. There exists a shared intellectual property agreement between the academic and philanthropic entities of the consortium for potential findings.

## Authors' contributions

SJE, PVC, MPV supervised the collection of gene expression data by using the Affymetrix Genechips. LT, VS, TC collected gene expression data by using the Illumina custom Beadchips. FM and SJW developed data management and analysis infrastructure and the Affymetrix custom CDF files. DW and WEB collected and compiled sample information that includes diagnosis, agonal stress and other clinical/demographic variables. MPV and WEB collected brain pH data. JL developed the analysis method in this work and performed the statistical analyses. JL and RMM wrote the manuscript. SJE, PVC, HT, MPV, WEB, EGJ, HA, SJW participated in data analysis and drafting of the manuscript. WEB, EGJ, HA, SJW, RMM conceived, coordinated, and supervised the overall study. All authors read and approved the final manuscript.

## Supplementary Material

Additional file 1 Technical batch effects. a. The color-coded correlation matrix among the same 201 AnCg samples as in Figure 1a, but calculated by using only the 68 control probe sets targeting spiked-in *E. coli *transcripts. Sample order was the same as in Figure 1a. b. Similar correlation heatmap based on 700-gene Illumina data. Samples were ordered by Cohort. c. Correlation matrix among 24 chips that represented eight samples ran three times each. The first time (samples 1–8 counting from lower left) was on U133A chips; the next two times were on U133_Plus2 chips. Shown are results after median centering of the chip-type blocks. The off-diagonal lines of high similarity are for the three replicate chips of the same samples, indicating that sample-sample differences were reproducibly measured across two chip types after removing the block effect. Click here for file

Additional file 2 Heatmap of normalized expression levels for "top 25%" genes in 201 AnCg samples. Shown are log-transformed, normalized expression levels of 3184 transcripts across 201 AnCg samples. These genes have the highest 25% of Type 1- Type 2 absolute t scores, and have been used to calculate the sample-sample correlations shown in Figure 3, upper left panel. The genes are ordered from left to right by their coefficients in the first principal component (i.e., each gene's "loading" in the first eigenvector), whereas the samples are ordered from top to bottom by their first principal component scores. The color scale is for log2 values of -3 (8-fold lower expression) to 3 (8-fold higher), with some values in the upper left corner being greater than 3. These saturated values were shown in brown. Click here for file

Additional file 3 Expression levels of 3841 most changed genes. Normalized expression levels for 3841 genes in 201 samples as shown in Additional File 2. Samples and genes are ordered by PC results, and formatted in Treeview format. Click here for file

Additional file 4 Cross-validation errors in classifying samples. Number of cross-validation errors as a function of number of genes used in the nearest Shrunken Centroid classification [25] where the 201 AnCg samples were analyzed, and the Type 1-Type 2 designations were taken as known. Lower panel showed the errors for Type 1 and Type 2 samples separately. Click here for file

Additional file 5 Comparison of Type 1-Type 2 differences across brain regions. Scatter plots of t scores for 12,734 transcripts on the U133A chips across six regions, showing that the Type-1 versus Type-2 comparisons in these brain regions are highly correlated. The t scores are calculated by comparing about 20% strongest Type 1 samples against about 20% strongest Type 2 samples in each region. The samples are ranked by a Principal Component Analysis by using all transcripts. Click here for file

Additional file 6 Most strongly changed genes upon agonal stress. The spreadsheet "1000Up_LowpH" listed 1000 most strongly up-regulated genes among the Type-2, low-pH samples and their t scores in five regions. The second spreadsheet "1000Down_LowpH" listed the corresponding 1000 most strongly down-regulated transcripts. The Unigene ID's are from Unigene Build 176. Click here for file
