# Supplementary figures and images for: Sample matching by inferred agonal stress in gene expression analyses of the brain
Source: BMC Genomics. 2007 Sep 24;8:336. doi: 10.1186/1471-2164-8-336 (PMC2213675; doi:10.1186/1471-2164-8-336)

A

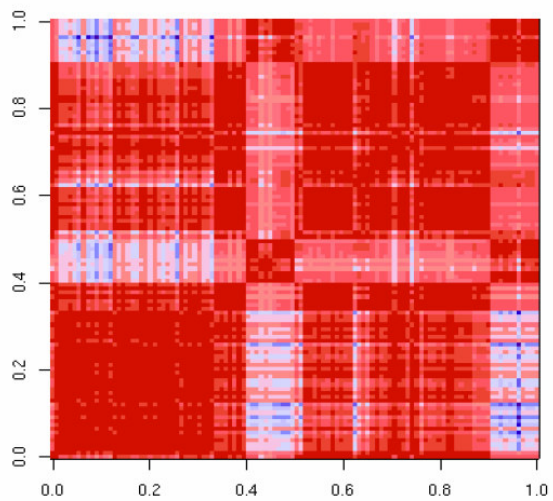

B

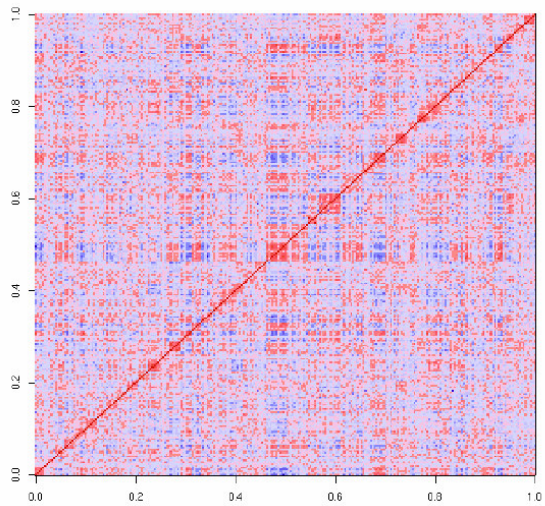

C

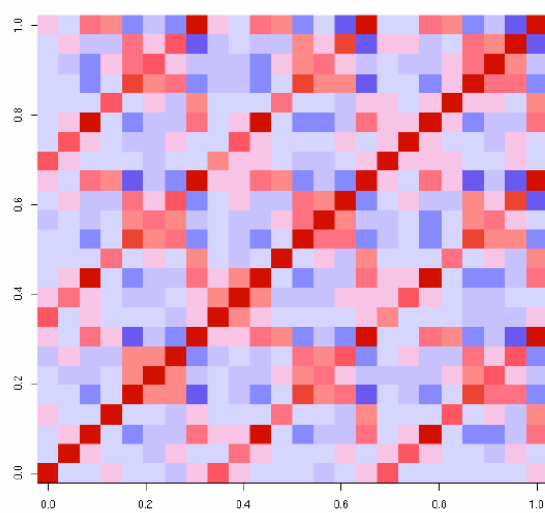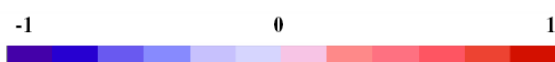

Supplement: Additional file 1 — Technical batch effects. a. The color-coded correlation matrix among the same 201 AnCg samples as in Figure 1a, but calculated by using only the 68 control probe sets targeting spiked-in E. coli transcripts. Sample order was the same as in Figure 1a. b. Similar correlation heatmap based on 700-gene Illumina data. Samples were ordered by Cohort. c. Correlation matrix among 24 chips that represented eight samples ran three times each. The first time (samples 1–8 counting from lower left) was on U133A chips; the next two times were on U133_Plus2 chips. Shown are results after median centering of the chip-type blocks. The off-diagonal lines of high similarity are for the three replicate chips of the same samples, indicating that sample-sample differences were reproducibly measured across two chip types after removing the block effect. [file 1471-2164-8-336-S1.pdf]

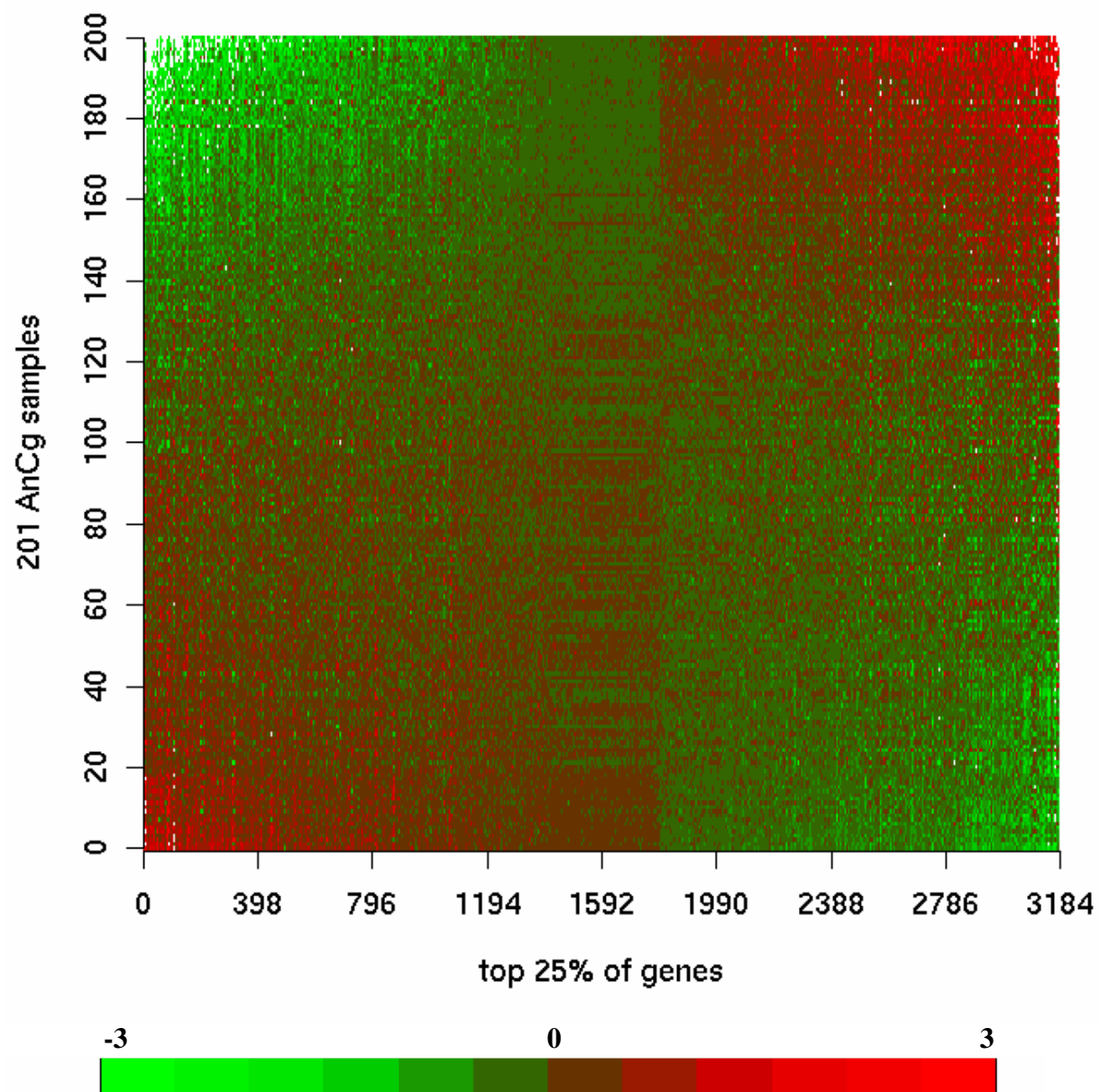

Supplement: Additional file 2 — Heatmap of normalized expression levels for "top 25%" genes in 201 AnCg samples. Shown are log-transformed, normalized expression levels of 3184 transcripts across 201 AnCg samples. These genes have the highest 25% of Type 1- Type 2 absolute t scores, and have been used to calculate the sample-sample correlations shown in Figure 3, upper left panel. The genes are ordered from left to right by their coefficients in the first principal component (i.e., each gene's "loading" in the first eigenvector), whereas the samples are ordered from top to bottom by their first principal component scores. The color scale is for log2 values of -3 (8-fold lower expression) to 3 (8-fold higher), with some values in the upper left corner being greater than 3. These saturated values were shown in brown. [file 1471-2164-8-336-S2.pdf]

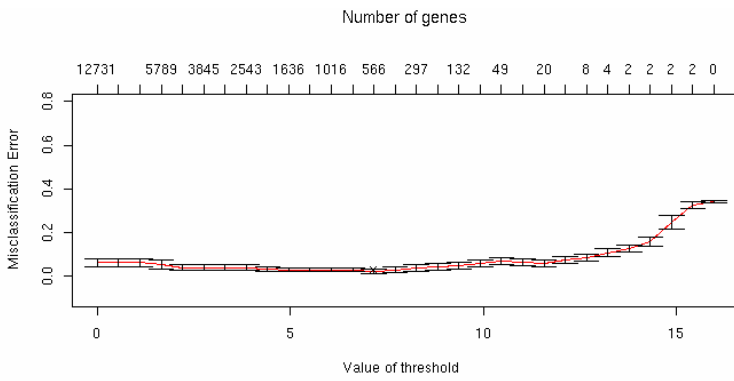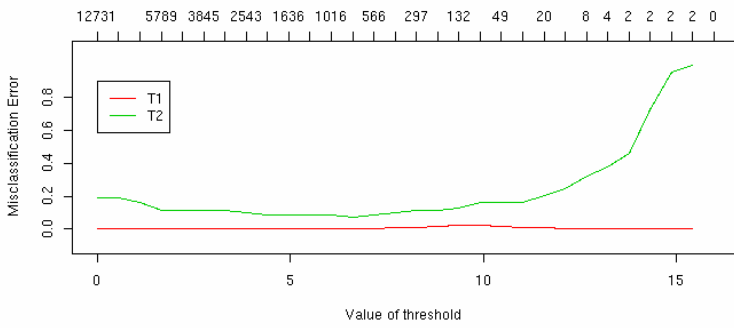

Supplement: Additional file 4 — Cross-validation errors in classifying samples. Number of cross-validation errors as a function of number of genes used in the nearest Shrunken Centroid classification [25] where the 201 AnCg samples were analyzed, and the Type 1-Type 2 designations were taken as known. Lower panel showed the errors for Type 1 and Type 2 samples separately. [file 1471-2164-8-336-S4.pdf]

Comparison of T1-T2 t scores between regions

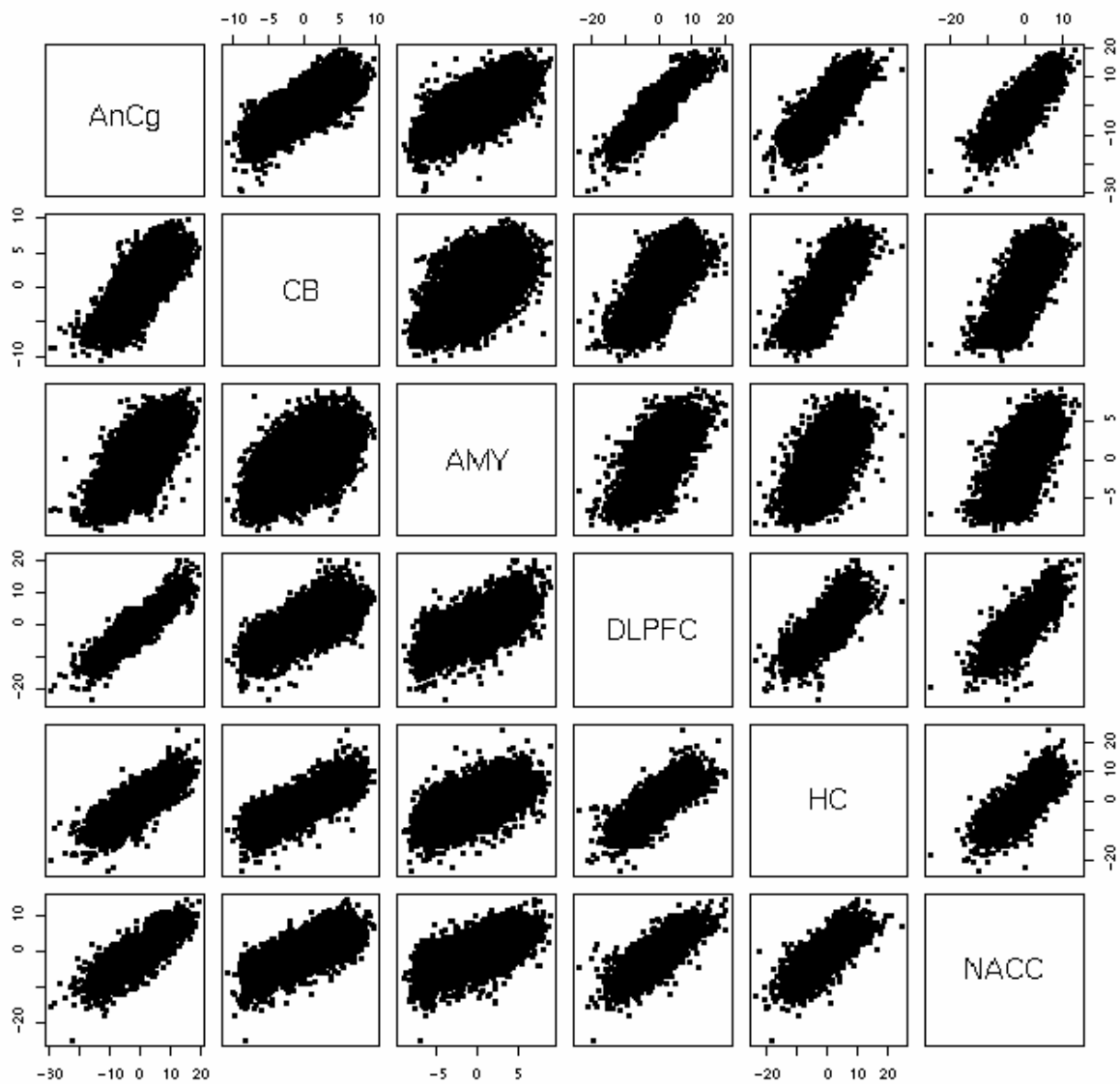

Supplement: Additional file 5 — Comparison of Type 1-Type 2 differences across brain regions. Scatter plots of t scores for 12,734 transcripts on the U133A chips across six regions, showing that the Type-1 versus Type-2 comparisons in these brain regions are highly correlated. The t scores are calculated by comparing about 20% strongest Type 1 samples against about 20% strongest Type 2 samples in each region. The samples are ranked by a Principal Component Analysis by using all transcripts. [file 1471-2164-8-336-S5.pdf]
